# Time variation of high-risk groups for liver function deteriorations within fluctuating long-term liver function after hepatic radiotherapy in patients with hepatocellular carcinoma

**DOI:** 10.1186/s40001-024-01692-z

**Published:** 2024-02-07

**Authors:** Yu-Lun Tsai, Pei-Chieh Yu, Hsin-Hua Nien, Tzu-Pin Lu

**Affiliations:** 1 Institute of Epidemiology and Preventive Medicine, College of Public Health, National Taiwan University, Taipei, Taiwan; 2 Department of Radiation Oncology, Cathay General Hospital, Taipei, Taiwan; 3 School of Medicine, China Medical University, Taichung, Taiwan; 4 School of Medicine, Fu Jen Catholic University, New Taipei City, Taiwan; 5 Institute of Biomedical Engineering, College of Electrical and Computer Engineering, National Yang Ming Chiao Tung University, Hsinchu, Taiwan; 6 Bioinformatics and Biostatistics Core, Center of Genomic and Precision Medicine, National Taiwan University, Taipei, Taiwan; 7 Institute of Health Data Analytics and Statistics, College of Public Health, National Taiwan University, Taipei, Taiwan

**Keywords:** Time variation, High-risk group, Long-term liver function, Hepatic radiotherapy, Hepatocellular carcinoma

## Abstract

**Purpose:**

The purpose of this study is to find essential risk factors associated with liver function (LF) deteriorations within fluctuating long-term LF and their time-varying effects in patients with hepatocellular carcinoma (HCC) receiving hepatic radiotherapy and to identify high-risk groups for adverse LF deteriorations and their changes over time in facilitating the prevention of hepatic decompensation and the improvement of survival.

**Materials and methods:**

A total of 133 HCC patients treated by hepatic radiotherapy were enrolled. A study design was conducted to convert posttreatment long-term LF with fluctuating levels over time to recurrent LF events using defined upgrades in a grading scale. The hazard ratios (HR) of pretreatment biochemical, demographic, clinical, and dosimetric factors in developing posttreatment LF events were estimated using the Cox model. Methodologies of the counting process approach, robust variance estimation, goodness-of-fit testing based on the Schoenfeld residuals, and time-dependent covariates in survival analysis were employed to handle the correlation within subjects and evaluate the time-varying effects during long-term follow-up.

**Results:**

Baseline LF score before radiotherapy and gender were significant factors. Initial HR in developing LF events was 1.17 (95% CI 1.11–1.23; *P* < 0.001) for each increase of baseline LF score and kept almost constant over time (HR, 1.00; 95% CI 1.00–1.01; *P* = 0.065). However, no difference was observed regarding initial hazards for gender (HR, 1.00; 95% CI 0.64–1.56; P = 0.994), but the hazard for women got higher monthly over time compared with men (HR, 1.04; 95% CI 1.01–1.07; *P* = 0.006).

**Conclusions:**

High-risk groups for adverse LF deteriorations after hepatic radiotherapy may change over time. Patients with poor baseline LF are vulnerable from the beginning. Women require prevention strategies and careful monitoring for deteriorations at a later stage.

**Supplementary Information:**

The online version contains supplementary material available at 10.1186/s40001-024-01692-z.

## Introduction

The liver is responsible for many metabolic functions in the human body. Clinicians are required to assess abnormal liver function (LF) daily [1]. The most common LF tests, also referred to as liver chemistries, typically include serum bilirubin (BIL), aspartate transaminase (AST), alanine transaminase (ALT), alkaline phosphatase (ALKP), gamma-glutamyl transferase, prothrombin time/international normalized ratio (INR), total protein, and albumin (ALB) [2]. Among these, the most frequently ordered tests in clinical practice are BIL, AST, ALT, and ALKP [1]. Hepatocellular injury is defined as a disproportionate elevation of AST and ALT levels, while cholestatic injury is characterized by a disproportionate elevation of ALKP [1]. Total BIL elevation can occur in either hepatocellular or cholestatic diseases [1]. In addition, hepatic synthetic function tests, specifically INR and ALB [2], which are encompassed in the Child–Pugh score, play a crucial role in assessing LF and radiation-induced liver disease (RILD) [3]. Special attention must always be paid, while LF is elevated. Abnormal LF is associated with a high risk for liver disease mortality and increased risk for all-cause mortality [4–7].

Hepatocellular carcinoma (HCC) has a wide range of variability in tumor growth and spread [8, 9]. Radiotherapy is one of the most critical modalities in treating unresectable HCC [10–12]. If appropriately applied, patients could have long-term survival, even for advanced HCC showing macroscopic vascular invasion, with a median overall survival exceeding a year [13]. However, abnormal LF may occur after radiotherapy. Former studies mainly focused on LF deteriorations within 3–4 months after receiving radiotherapy or alleged RILD [3, 14, 15]. Historically, classical RILD was characterized by anicteric hepatomegaly, ascites, and an isolated elevation in ALKP, but has become rare in the current era with advanced radiotherapy technology [3, 14]. Non-classic RILD is now much more common, presenting as a general decline in LF, markedly elevated transaminases, and jaundice [3, 14]. Pretreatment factors predicting posttreatment RILD have been reported, encompassing biochemical [16, 17], clinical [16, 17], and dosimetric factors [18, 19]. The Child–Pugh score is significantly associated with RILD, revealing a relative risk or odds ratio ranging from 3.7 to 13.6 in Child–Pugh grade B patients compared with grade A patients [16, 17]. Hepatitis B virus (HBV) status strongly predicts RILD development, with HBV carriers having an estimated odds ratio of 9.3 compared with non-carriers [17]. Regarding dosimetric factors, a significant cutoff value was reported for the normal liver receiving a radiation dose of more than 30 Gy [18, 19]. Posttreatment RILD may have a negative impact on survival [20]. Short-term RILD is relatively better understood currently.

Beyond the scope of short-term outcomes, abnormal LF may be found in long-term survivors during the first 2 years after radiotherapy, especially in patients with pre-existing liver damage [21]. Long-term follow-up longitudinal data studies are more relevant to understanding the whole picture of changeable LF and identifying vulnerable groups for LF deteriorations and their changes over time. However, well-designed studies are lacking, and the implementation of such studies is complex, because LF has complicated collectives that consist of multiple biomarkers with individual clinical meanings [1–3]. The presentation of each type of LF during long-term follow-up is ever-changing with high variability.

In this study, we aim to conduct a study design overcoming the above difficulties, to find essential risk factors associated with LF deteriorations within fluctuating long-term LF over a year after hepatic radiotherapy, to evaluate the time-varying effects of the factors for high-risk group identification over time, and finally to prevent adverse LF deteriorations in daily clinical practice.

## Methods and materials

The study primarily set out to find essential risk factors associated with LF deteriorations and their time-varying effects within fluctuating long-term LF. This objective was problematic using a simple methodology, because fluctuating LF was not a linear outcome with a regular trend. A single patient may experience several episodes of LF deterioration during long-term follow-up.

We conducted a study designed to convert numeric LF levels to recurrent LF events, a particular form of time-to-event data called recurrent event data in survival analysis. We also derived our estimates using the counting process approach and robust variance estimation to handle the dependence of recurrent events within a subject.

### Study population

**Fig. 1:**
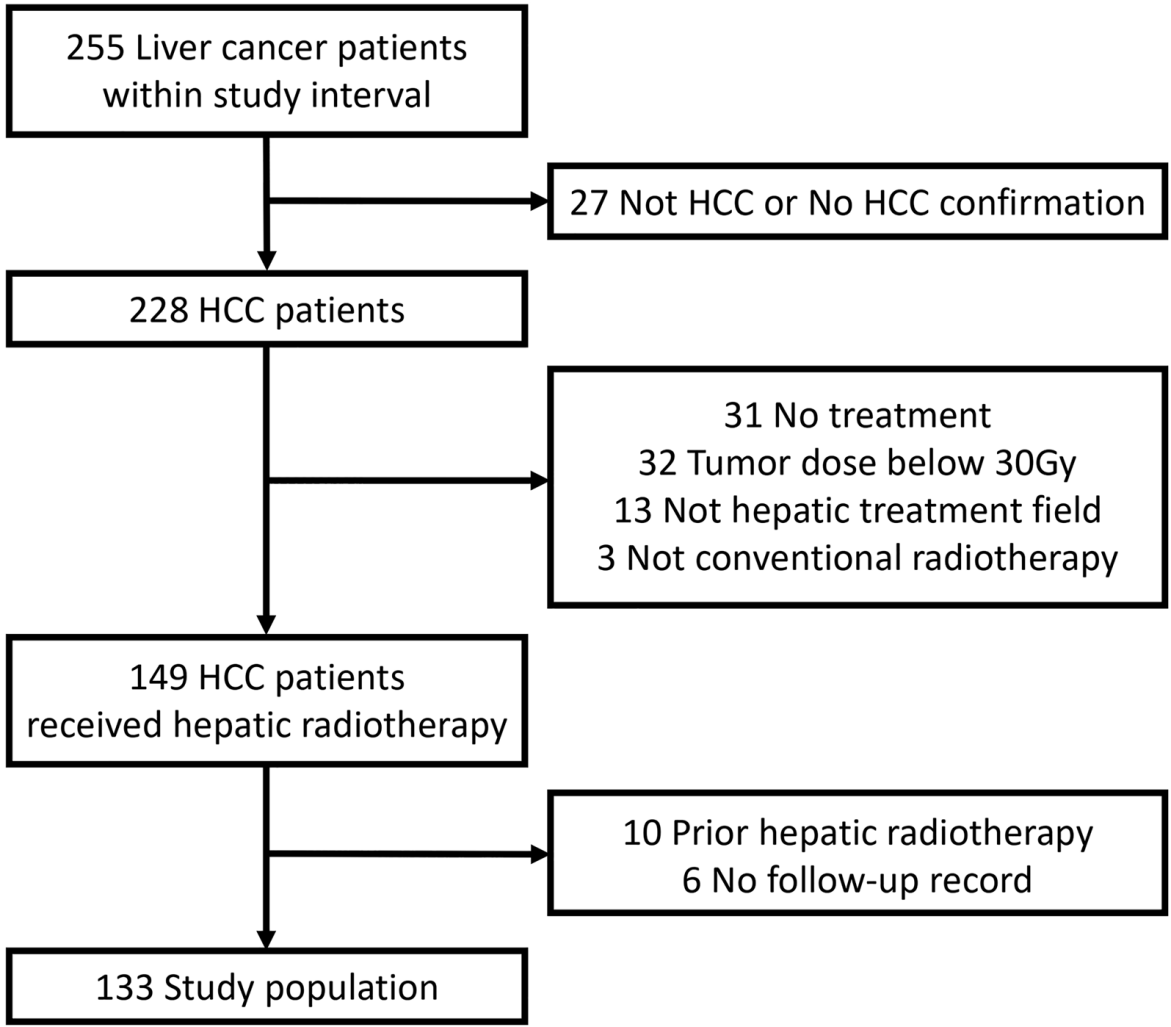
Flow chart of the study population selection process

With the ethical approval of an institutional review board, all patients with HCC treated by conventional hepatic radiotherapy at our institution between August 2013 and June 2021 were considered for this study. A total of 133 patients who met our study criteria were enrolled. Figure 1 illustrates the process of selection for the study population. The diagnosis of HCC was proved by histology or based on the radiology criteria [22, 23]. The hepatic radiotherapy could be liver-directed irradiation or treatment for macroscopic vascular invasion, including portal vein tumor thrombosis (PVTT) and inferior vena cava tumor thrombosis (IVCTT) with radiation doses to the liver. Eligible patients should complete the treatment course or receive a tumor dose above 30 Gy. Patients who had received prior hepatic radiotherapy or had no follow-up record after treatment were excluded. No participants were scheduled for liver transplantation or resection after radiotherapy.

### Data collection and processing

Biochemical, demographic, clinical, and dosimetric baseline characteristics of the patients were collected. Baseline LF was defined as the LF within 1 month prior to radiotherapy. We gathered the levels of six common serum liver chemistries (i.e., total BIL, AST, ALT, ALKP, INR, and ALB) representing LF status [1–3, 24]. Baseline individual LF scores before radiotherapy were then calculated according to the grade conversion of LF levels using a grading scale with descriptions of the grades corresponding to Common Terminology Criteria for Adverse Events version 5.0 (CTCAE v5.0) with slight adjustment [25]. The cutoff values were, respectively, 1 × upper limit of normal (ULN), 1.5 × ULN, 3 × ULN, and 10 × ULN for BIL score (score 0: ≤ 1.5 mg/dL; score 1: > 1.5–2.25 mg/dL; score 2: > 2.25–4.5 mg/dL; score 3: > 4.5–15 mg/dL; score 4: > 15 mg/dL), 1 × ULN, 3 × ULN, 5 × ULN, and 20 × ULN for AST score (score 0: ≤ 35 IU/L; score 1: > 35–105 IU/L; score 2: > 105–175 IU/L; score 3: > 175–700 IU/L, score 4: > 700 IU/L), 1 × ULN, 3 × ULN, 5 × ULN, and 20 × ULN for ALT score (score 0: ≤ 35 IU/L; score 1: > 35–105 IU/L; score 2: > 105–175 IU/L; score 3: > 175–700 IU/L, score 4: > 700 IU/L), 1 × ULN, 2.5 × ULN, 5 × ULN, and 20 × ULN for ALKP score (score 0: ≤ 120 IU/L; score 1: > 120–300 IU/L; score 2: > 300–600 IU/L; score 3: > 600–2400 IU/L, score 4: > 2400 IU/L), 1.2, 1.5, and 2.5 for INR score (score 0: ≤ 1.2; score 1: > 1.2–1.5; score 2: > 1.5–2.5; score 3: > 2.5), and 3.5, 3, and 2 g/dL for ALB score (score 0: ≥ 3.5 g/dL; score 1: < 3.5–3 g/dL; score 2: < 3–2 g/dL; score 3: < 2 g/dL). The total score was the sum of individual scores (ALL6 score: the sum of BIL, AST, ALT, ALKP, INR, and ALB scores).

The age at treatment and gender of patients were recorded. Age was divided into four groups by quartiles. The presence of PVTT and IVCTT was determined through computed tomography (CT) or magnetic resonance imaging images, indicating the severity of tumor invasion. The prevalence of HBV and hepatitis C virus (HCV) infection is high in the Asian population. We also applied the test results for hepatitis B surface antigen (HBsAg) and hepatitis C antibody (Anti-HCV), denoting HBV and HCV infections.

### Hepatic radiotherapy

The treatment was conventional photon–beam radiotherapy delivered by a linear accelerator (Truebeam STx, Varian Medical Systems, Palo Alto, CA, USA) with a beam energy of 6 or 10 MV. The techniques involved either intensity-modulated radiation therapy or volumetric-modulated arc therapy, with or without image guidance delivery. The prescribed doses were 30–60 Gy, with a fraction of 1.8–3 Gy. The treating physician determined the clinical target volume (CTV) based on the lesion observed in the imaging of CT simulation, with considerations for respiratory motion on four-dimensional CT (4DCT) if available. If 4DCT is not available, the determination was made based on the physician's concerns regarding respiratory motion concerning tumor location [26]. The planning target volume (PTV) was then derived with an additional expansion ranging from 0.5 to 1 cm. The primary dose constraints for treatment planning aimed to attain the following criteria: PTV *D*_95%_ ≥ 95% of the prescribed dose, spinal cord max dose < 45 Gy, normal liver mean dose < 30 Gy and *D*_30%_ < 27 Gy, bilateral kidneys *D*_33%_ < 20 Gy and *D*_67%_ < 18 Gy, stomach *D*_33%_ < 60 Gy and *D*_67%_ < 55 Gy, and bowel bag *V*_45Gy_ < 150 cc. Plan delivery quality was assessed under the 3%/3 mm criterion of gamma analysis, and portal dosimetry was passed for all treatment plans. The tolerance limits were set at 97% for the passing rate of area gamma < 1, 3 for the maximum gamma, and 0.5 for the average gamma, respectively.

Dosimetric factors were extracted from the treatment planning system for further study, including CTV, normal liver volume (NLV), and normal liver mean dose (NLD_mean_) [27, 28]. The units in the presentation were mL and Gy, respectively.

### Follow-up

Patients were followed up after radiotherapy intensively, with intervals no longer than every 3–6 months for 2 years and then every 6–12 months per institutional cancer treatment surveillance guidelines. Routine follow-up items contained clinical, blood, and imaging examinations. For this study, person-months were calculated from the date of completion of radiotherapy to the last date of visit with LF tests of interest before death, loss to follow-up, study end, and second course of hepatic radiotherapy if applied. Studied outcomes were all available results of the LF test for BIL, AST, ALT, ALKP, INR, and ALB during accounted person-months. LF levels and dates of the tests were recorded.

### Definitions of LF events

Posttreatment LF levels were converted to grades according to the aforementioned grading scale, the same for the pretreatment baseline LF score. LF events were defined as LF deteriorations with an upgrade during follow-up. Individual LF events (BIL, AST, ALT, ALKP, INR, and ALB events) were identified through an upgrade of the LF of interest. Combined LF events (ALL6 events) were recognized at the time of any individual events. For the first visit with the LF test of interest, the upgrade was compared with the same LF test within 1 week before the completion of radiotherapy. The rest of the visits were compared with the last visits. LF events could occur within only 1 type of LF that day (single events) or more types (synchronous events). LF events could be either a 1-grade LF upgrade (1-grade events) or > 1-grade LF upgrade (> 1-grade events). Data were censored after the final visit with the LF test of interest. The dates of censoring for different types of LF for a patient may be different. The above definitions converted numeric LF outcomes with fluctuating levels over time to recurrent events, a particular form of time-to-event data called recurrent event data. The outcome events might occur more than once for a given subject [29].

### Statistical analysis

The overall analytical method was survival analysis [29]. The covariates were input in the Cox proportional hazards (PH) model to estimate the hazard ratio (HR) of each covariate in developing LF events and to identify important risk factors by their statistical significance. In the model, baseline LF score before radiotherapy, age groups, CTV, NLV, and NLD_mean_ were treated as continuous variables. In contrast, gender, PVTT/IVCTT, HBV, and HCV were treated as categorical variables. The type of baseline LF score used aligned with the specific LF event of interest. For instance, when predicting the LF endpoint as ALL6 events, the baseline LF score input in the model was also the ALL6 score. Because the data form was recurrent event data, additional required methodologies were applied. The counting process approach was carried out to handle the recurrent event outcomes by separating time intervals at event times. In addition, robust variance estimation was used to adjust the estimated variances of regression coefficients to account for the misspecification of the assumed correlation structure. The robust variances, therefore, allowed tests of hypotheses and confidence intervals about model parameters that account for correlation within subjects [29].

PH assumption of the Cox model was tested using the goodness-of-fit testing approach based on the Schoenfeld residuals [29, 30]. For factors in which the PH assumption of the Cox PH model was violated, as indicated by the probability of the assumption of constant hazard over time being valid lower than 5%, but with significant main effects in the Cox PH model, we introduced time-dependent covariates. These covariates involved cross-products of the factors with the time variable in the extended Cox model. This adjustment facilitated a valid estimation of the initial HR and a better understanding of its trend over time [29].

The statistical calculations were applied in R, version 4.1.3, a programming language and software environment for statistical computing and graphics supported by the R Foundation for Statistical Computing. A *P* value < 0.05 was considered significant.

### Sensitivity analyses

Sensitivity analysis involves systematically repeating the statistical analysis, using different assumptions each time, to assess how sensitive the statistics are to changes in the analysis assumptions [31]. We performed a sensitivity analysis that synchronously substituted both the components of total LF score before radiotherapy and LF event from the six liver chemistries to the common three for non-classic RILD (ALL3 score and event: BIL, AST, and ALT). We also performed the sensitivity analysis for the common four for classic and non-classic RILD (ALL4 score and event: BIL, AST, ALT, and ALKP). We applied another sensitivity analysis, multiplying the cutoff values by a parameter from 0.7 to 1.3 by 0.1 each time and repeated the analysis to see how sensitive the effect estimates are to changes in different cutoff values of the LF grading scale.

## Results

### Characteristics of the study population

For the 133 eligible HCC patients being our study subjects, we recorded 1,627 person-months of follow-up. Table 1 shows the characteristics of the patients at baseline. Most patients were male (74.4%), and the median age at treatment was 68 years. Overall, patients with grade 0–1 baseline LF regarding individual liver chemistry accounted for the majority. Approximately 75.2% of patients referred for hepatic radiotherapy had PVTT or IVCTT. More than half of the patients (56.3%) demonstrated HBV infection, and about one-third (30.1%) showed HCV infection. Patients with viral hepatitis were treated with antiviral medications. Concerning dosimetric factors, patients had an average CTV of 298.5 mL (median 147.6 mL). The average NLV was 1365.2 mL (median 1216.9 mL) with a mean NLD_mean_ of 17.7 Gy (median 18.0 Gy) being delivered.

**Table 1:** Biochemical, demographic, clinical, and dosimetric characteristics of the patients at baseline and the person-months of follow-up

Characteristics	No. of patients (%)	Person-months
Baseline liver function score before radiotherapy		
Bilirubin (BIL)		
0	90 (67.6)	1285.2
1	17 (12.8)	217.0
2	21 (15.8)	117.4
3	4 (3.0)	5.6
4	1 (0.8)	1.8
Aspartate aminotransferase (AST)		
0	28 (21.0)	574.6
1	83 (62.4)	897.0
2	14 (10.5)	124.9
3	6 (4.5)	15.4
4	1 (0.8)	12.8
Missing	1 (0.8)	2.3
Alanine aminotransferase (ALT)		
0	59 (44.4)	788.4
1	65 (48.8)	734.0
2	8 (6.0)	91.8
3	1 (0.8)	12.8
4	0 (0.0)	0
Alkaline phosphatase (ALKP)		
0	59 (44.3)	983.7
1	45 (33.8)	324.5
2	15 (11.3)	75.0
3	1 (0.8)	14.9
4	0 (0.0)	0
Missing	13 (9.8)	228.9
International normalized ratio (INR)		
0	103 (77.4)	1404.3
1	17 (12.8)	110.7
2	2 (1.5)	7.3
3	0 (0.0)	0
Missing	11 (8.3)	104.7
Albumin (ALB)		
0	53 (39.8)	803.8
1	38 (28.6)	450.2
2	28 (21.1)	196.0
3	0 (0.0)	0
missing	14 (10.5)	177.0
Age		
≤ 60	35 (26.3)	446.1
> 60–68	32 (24.1)	353.9
> 68–76	35 (26.3)	538.0
> 76	31 (23.3)	289.0
Gender		
Female	34 (25.6)	422.6
Male	99 (74.4)	1204.4
Portal vein tumor thrombosis (PVTT)/Inferior vena cava tumor thrombosis (IVCTT)		
Positive	100 (75.2)	1049.7
Negative	33 (24.8)	577.3
Hepatitis B virus (HBV)		
Positive	75 (56.3)	814.5
Negative	55 (41.4)	724.3
Missing	3 (2.3)	88.2
Hepatitis C virus (HCV)		
Positive	40 (30.1)	627.6
Negative	90 (67.6)	911.2
Missing	3 (2.3)	88.2
Dosimetric factors of radiotherapy		
Clinical target volume (CTV)—mL		
Mean (SD)	298.5 (415.4)	–
Median (range)	147.6 (7.9 – 3316.7)	–
Normal liver volume (NLV)—mL		
Mean (SD)	1365.2 (638.5)	–
Median (range)	1216.9 (493.6 – 5517.6)	–
Normal liver mean dose (NLD_mean_)—Gy		
Mean (SD)	17.7 (4.8)	–
Median (range)	18.0 (2.2 – 29.7)	–
Total	133	1627.0

### LF events

Within the 1,627 person-months of follow-up, we observed 947 combined events of the six common liver chemistries that were recognized as corresponding to 1,416 individual events. Different types of individual events may occur on the same day, namely, synchronous events. Therefore, the sum of individual events was more than the number of combined events (for more details on the combinations, please see Additional file 3: Table S1 in the supplementary materials). Table 2 summarizes the numbers and percentages of LF events. BIL, AST, and ALT events were the most, followed by INR and ALB events, and ALKP events were the rarest. From the viewpoint of individual events, more than half were synchronous events except ALB events, and ≥ 80% were 1-grade events. However, from the viewpoint of combined events, single and 1-grade events were > 60%.

**Table 2:** Numbers and percentages of liver function (LF) events according to LF types

Type of liver function	Single event (%)	Synchronous event (%)	1-grade event (%)	> 1-grade event (%)	Total
BIL	148 (45.0)	181 (55.0)	263 (80.0)	66 (20.0)	329
AST	132 (37.0)	225 (63.0)	294 (82.4)	63 (17.6)	357
ALT	113 (37.5)	188 (62.5)	247 (82.1)	54 (17.9)	301
ALKP	33 (35.1)	61 (64.9)	87 (92.6)	7 (7.4)	94
INR	76 (49.4)	78 (50.6)	138 (89.6)	16 (10.4)	154
ALB	123 (68.0)	58 (32.0)	156 (86.2)	25 (13.8)	181
ALL6	625 (66.0)	322 (34.0)	583 (61.6)	364 (38.4)	947

Single events indicate that the events occurred within only 1 type of LF that day (synchronous events indicate more types). 1-grade events indicate the events were identified because of 1-grade LF deterioration

*BIL* bilirubin, *AST *aspartate aminotransferase, *ALT *alanine aminotransferase; *ALKP *alkaline phosphatase, *INR *international normalized ratio, *ALB *albumin, *ALL6 *bilirubin, aspartate aminotransferase, alanine aminotransferase, alkaline phosphatase, international normalized ratio, albumin

**Fig. 2:**
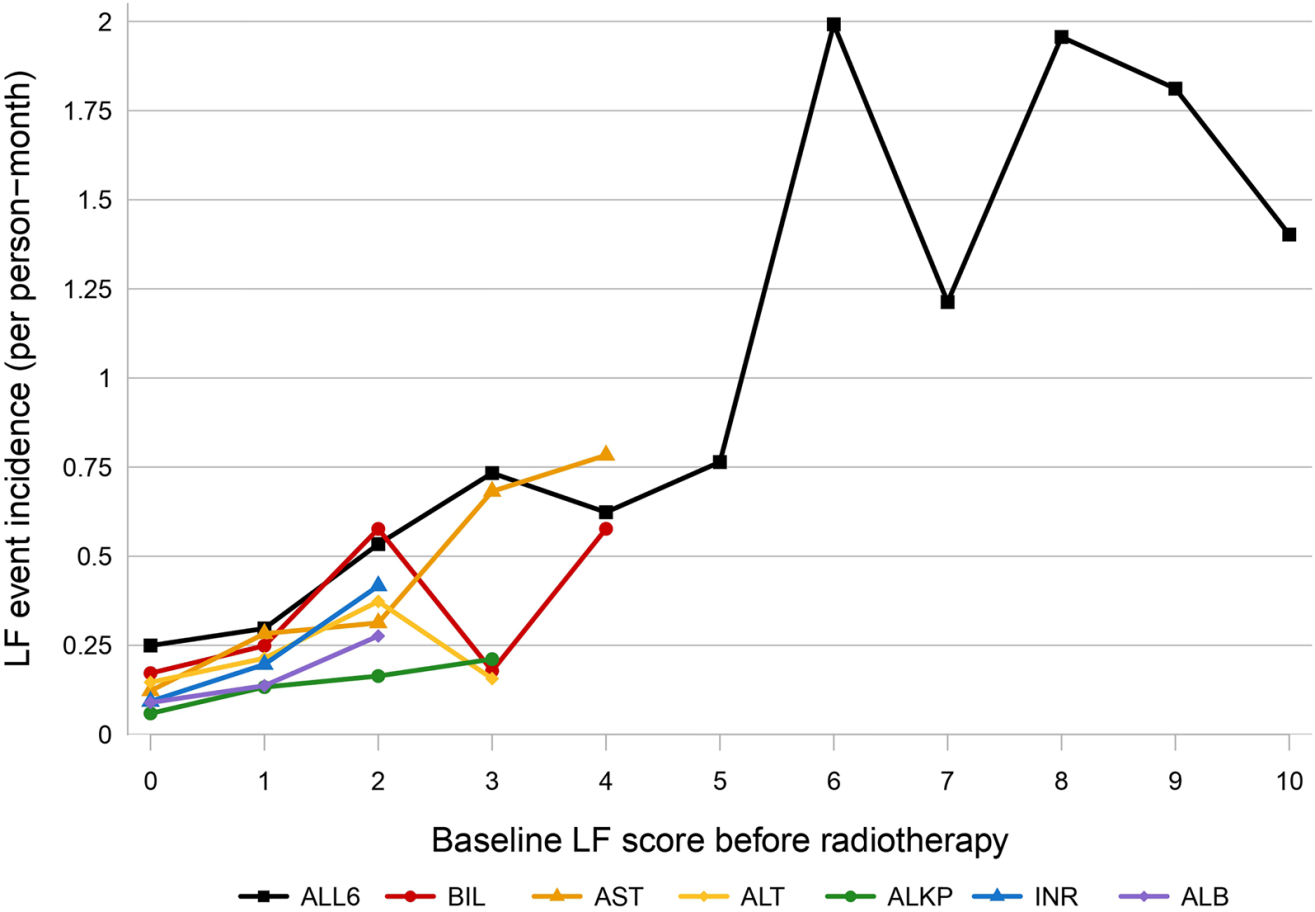
Baseline liver function (LF) score before radiotherapy correlated with the incidence rate of LF events after radiotherapy. Specifically, individual scores (BIL, AST, ALT, ALKP, INR, and ALB scores) aligned with LF grading levels in CTCAE v5.0. The total score is the sum of individual scores, referred to as the ALL6 score, which encompasses the cumulative values of BIL, AST, ALT, ALKP, INR, and ALB scores

As shown in Fig. 2, we discovered a concordant trend between the baseline LF score before radiotherapy and the incidence rate of LF events after radiotherapy. In general, the greater the score, the higher the incidence. All individual event incidences were < 0.25 per person-month for baseline LF scores of 0 and climbed up to > 0.75 per person-month for an AST score of 4. Combined event incidence started from 0.25 per person-month for a score of 0 and was almost up to 2 per person-month for a score of 6 and above.

**Fig. 3:**
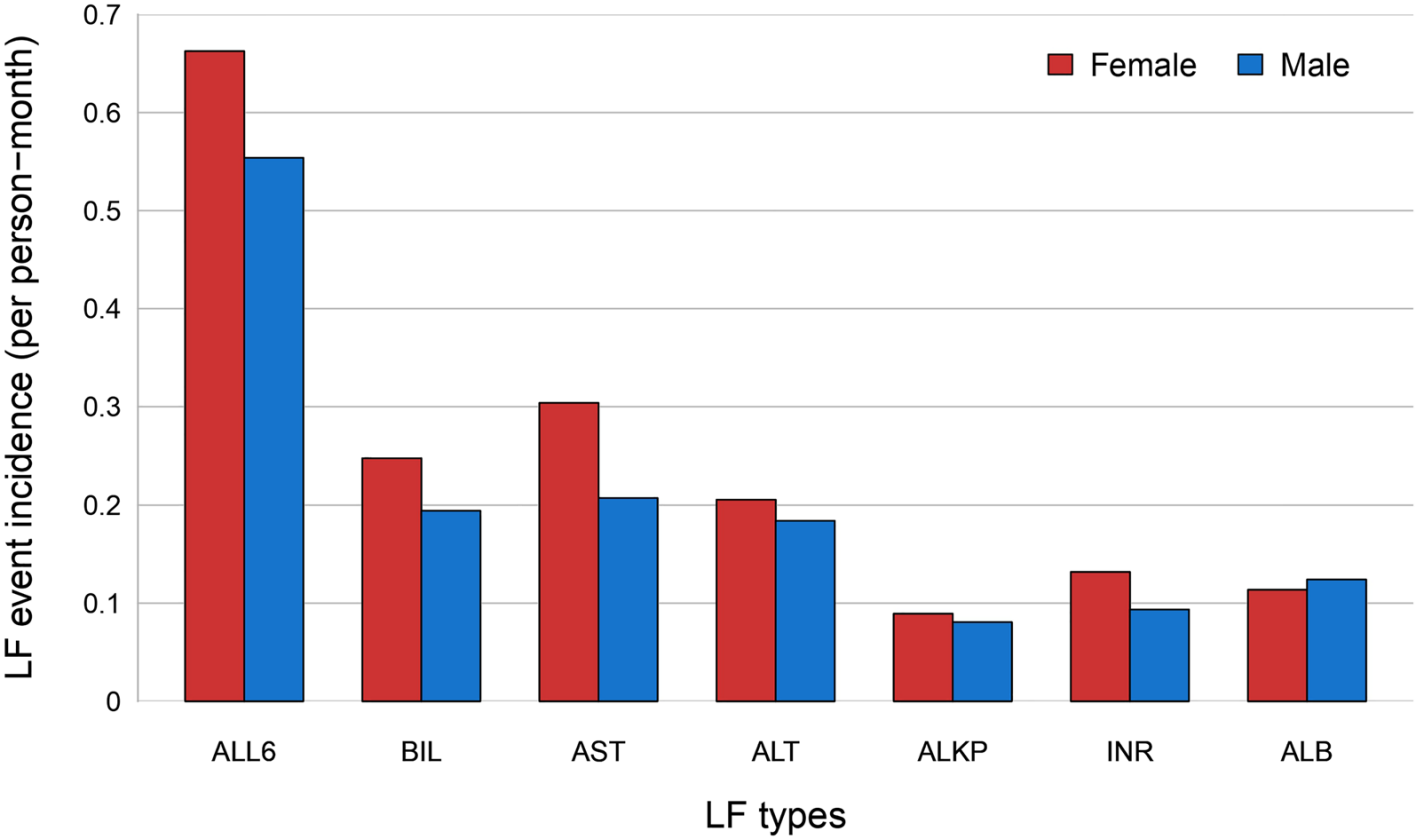
Women had a higher incidence rate of liver function (LF) events versus men for all types of LF, only except for albumin

Figure 3 depicts the incidence rate of LF events based on gender. Women had higher incidences of combined events and individual events for BIL, AST, ALT, ALKP, and INR comprehensively. The difference in the incidence of combined events was around 0.1 per person-month, approximately 18% of the male incidence. AST had the most significant difference for individual events, around 0.1 per person-month, about 50% of the male AST incidence. ALB was the only one with a slightly higher incidence for men.

### Hazard ratios in developing LF events

The upper portion of Table 3 demonstrates the estimated HRs of analyzed covariates in developing combined LF events in the Cox PH model. Baseline total LF score before radiotherapy (ALL6 score) and gender were the covariates with statistically significant *P* values (< 0.001 and 0.046, respectively), which could be crucial LF-event risk factors. The estimated HRs were 1.21 (95% CI 1.16–1.27) for each increase of the ALL6 score and 1.44 (95% CI 1.01–2.06) for women versus men. In the goodness-of-fit test based on the Schoenfeld residuals, both the ALL6 score and gender violated the PH assumption (please see Additional file 4: Table S2 in the supplementary materials). We, therefore, introduced time-dependent covariates for both in the extended Cox model, and the results are shown in the lower portion of Table 3. ALL6 score held the increased initial HR (1.17; 95% CI 1.11–1.23; *P* < 0.001) right after radiotherapy (time 0) for each increase of the score and was kept almost constant yet slightly increased over time (HR, 1.00; 95% CI 1.00–1.01; *P* = 0.065). On the other hand, women had equivalent initial hazard as men right after radiotherapy (HR, 1.00; 95% CI 0.64–1.56; *P* = 0.994), but got higher monthly over time (HR, 1.04; 95% CI 1.01–1.07; *P* = 0.006).

**Table 3:** Hazard ratios in developing combined liver function (LF) events for the six common liver chemistries with/without introducing time-dependent covariates in the Cox models

Model	Covariate	Value	Hazard ratio (95% CI)	*P* value
ALL6 score + covariates	ALL6 score	0, 1, 2, 3, 4, 5, 6, 7, 8, 9, 10 (cont.)	**1.21 (1.16 – 1.27)**	< 0.001*
	Age	≤ 60, > 60–68, > 68–76, > 76 (cont.)	0.96 (0.83 – 1.11)	0.583
	Gender	Female (Male ref.)	**1.44 (1.01 – 2.06)**	0.046*
	PVTT/IVCTT	Positive (Negative ref.)	1.33 (0.93 – 1.91)	0.114
	HBV	Positive (Negative ref.)	1.03 (0.73 – 1.45)	0.868
	HCV	Positive (Negative ref.)	1.01 (0.67 – 1.52)	0.961
	CTV	Per 100 mL (cont.)	0.98 (0.95 – 1.02)	0.376
	NLV	Per 100 mL (cont.)	1.00 (0.98 – 1.02)	0.920
	NLD_mean_	Per 1 Gy (cont.)	0.98 (0.95 – 1.00)	0.090
ALL6 score + covariates + time-dependent covariates	ALL6 score	0, 1, 2, 3, 4, 5, 6, 7, 8, 9, 10 (cont.)	**1.17 (1.11 – 1.23)**	< 0.001*
	Age	≤ 60, > 60–68, > 68–76, > 76 (cont.)	0.96 (0.83 – 1.10)	0.543
	Gender	Female (Male ref.)	1.00 (0.64 – 1.56)	0.994
	PVTT/IVCTT	Positive (Negative ref.)	1.31 (0.93 – 1.85)	0.125
	HBV	Positive (Negative ref.)	1.10 (0.79 – 1.54)	0.560
	HCV	Positive (Negative ref.)	1.04 (0.69 – 1.56)	0.856
	CTV	Per 100 mL (cont.)	0.98 (0.95 – 1.02)	0.375
	NLV	Per 100 mL (cont.)	1.00 (0.98 – 1.02)	0.853
	NLD_mean_	Per 1 Gy (cont.)	0.98 (0.95 – 1.01)	0.171
	ALL6 score × time	0, 1, 2, 3, 4, 5, 6, 7, 8, 9, 10 (cont.) per 1 month	1.00 (1.00 – 1.01)	0.065
	Gender × time	Female (Male ref.) per 1 month	**1.04 (1.01 – 1.07)**	0.006*

The hazard increases for baseline total LF score increases (ALL6 score) are kept almost constant over time. Women had equivalent initial hazard as men right after radiotherapy but got higher monthly over time

*ALL6 *bilirubin, aspartate aminotransferase, alanine aminotransferase, alkaline phosphatase, international normalized ratio, albumin, *PVTT *portal vein tumor thrombosis, *IVCTT* inferior vena cava tumor thrombosis, *HBV *hepatitis B virus, *HCV *hepatitis C virus, *CTV *clinical target volume, *NLV *normal liver volume, *NLD*_*mean*_ normal liver mean dose

*Statistical significance

Considering the development of individual LF events, a higher baseline individual LF score by the type of LF event of interest, females, and PVTT/IVCTT had the potential to increase HRs in general (please see Additional file 5: Table S3 in the supplementary materials). The HRs ranged from 1.40 to 1.99 for each increase of individual score with statistical significance for all types of LF. Significant HRs for gender were 1.65 for AST, 1.62 for ALT, and 2.34 for INR events. In addition, significant HRs for PVTT/IVCTT were, respectively, 1.50 for AST and 2.33 for ALB events.

### Sensitivity analyses

In the sensitivity analysis that substituted the LF components from six liver chemistries to three or four, we still discovered an increasing trend of LF event incidence along with an increased baseline LF score before radiotherapy (please see Additional file 1: Figure S1 and Additional file 2: Figure S2 in the supplementary materials). Initial HR still significantly increased after radiotherapy and with a slight increase over time. The estimations of HR were almost the same (for more details on the models, please see Additional file 6: Table S4 in the supplementary materials). In another sensitivity analysis changing cutoff values of the LF grading scale, the variations of the HRs for ALL6 score, gender, and time-dependent covariates were minor compared to original estimations (please see Additional file 7: Table S5 in the supplementary materials). These findings affirm the stability and reliability of the results.

## Discussion

The study results generally indicate that higher baseline LF scores before radiotherapy and females would be significant risk factors for predicting posttreatment LF deteriorations within fluctuating long-term LF. Because the features of these two factors leading to LF events are different, high-risk groups for LF deteriorations that should be taken care of during long-term follow-up may change over time. All patients with pretreatment liver dysfunction are vulnerable to posttreatment LF re-upgrading starting from the completion of radiotherapy. On the other hand, although not different from men at the beginning after treatment, women must carefully monitor their LF later in a longer follow-up. This finding is new and surprising, as patients with different hazards for LF deteriorations after radiotherapy depend on their gender. Previous studies have indicated that sex hormones and sex hormone-binding globulin are associated with LF, liver fat, and liver disease [32, 33]. Estrogen and estrogen receptor alpha may contribute to fatty liver and chronic liver diseases in women [34–36]. Furthermore, sex-dependent differences in cholestasis have also been reported [37]. Sex hormones emerge as a worthy candidate for further study to support our findings. Moreover, if more extensive studies can corroborate this observation, distinct prevention strategies and monitoring protocols should likely be considered for female populations with HCC receiving radiotherapy.

Applying pretreatment LF score to predict posttreatment LF status is consistent with former study results for RILD. Combined LF scores, such as the Child–Pugh score [16, 38], the model for end-stage liver disease score [39], and the albumin–bilirubin score [40–42], demonstrated usefulness in assessing the risk of RILD within 3–4 months after radiotherapy in either conventional or ablative radiotherapy. We broadened the utility of the baseline LF score in the present study to predict the upgrade of LF with the meanings in temporality and severity during long-term follow-up. In addition, the total score we used can be divided into individual scores to evaluate individual LF deteriorations of interest.

For other covariates without statistical significance in the model under a significant level of 0.05, it does not mean they are definitely without potential associated with long-term LF. Patients with PVTT or IVCTT yielded an HR of 1.31 to develop combined LF events by our estimation compared with those without. This effect size is large enough and worthy of further attention. Moreover, PVTT/IVCTT showed significant HRs in the individual event models for AST and ALB. Regarding viral hepatitis, previous studies found that HBV carriers have a greater susceptibility to RILD [17]. A high HBV reactivation rate probably induces this phenomenon from a bystander effect on irradiated endothelial cells releasing cytokines [43, 44]. However, antiviral treatments are currently involved in our national public health insurance program. Many HBV and HCV patients enrolled in the study had a serum level of undetectable or few viral nucleic acids, which may lead to an equivalent LF deterioration hazard as uninfected participants. As for dosimetric factors, because physicians have to prescribe a target dose, delineate CTV, and preserve normal liver in treatment planning according to much clinical information, anonymous information will subsequently confound the effect estimation of the factors, likely confounding by indication in epidemiology. To better understand the effects of the dosimetric factors on LF, prospective blinded studies are required.

While we try to identify high-risk groups for LF deteriorations after radiotherapy, other researchers try to mitigate LF damage using another approach [45]. LF biomarkers and functional liver imaging, such as indocyanine green and [99mTc]-sulfur colloid single photon emission computed tomography/computed tomography, were shown to be early detectors in assessing hepatotoxicity [46, 47]. Therapeutic agents and drugs may reduce the extent and incidence of liver injury [48]. For example, a prospective randomized trial examined the preventive effects of a combined regimen consisting of pentoxifylline, ursodeoxycholic acid, and low-dose low molecular-weight heparin [49]. Local and global function models of the liver from physics contribution could improve the evaluation of regional dose responses of LF and guidance for individualized treatment planning [50]. The evolution of machine learning elaboration has potential in anatomical structure detections [51], automated segmentations [52], and outcome predictions [53]. This computer science may help clinicians make decisions from complex data through statistical models.

This study offers some advantages. First, it is the first study to delve into long-term LF after radiotherapy. Managing fluctuating long-term LF with high variability is a challenge, and we have defined the LF upgrades through clear definitions that correspond to clinical meanings. Second, the results reveal that gender is a risk factor for LF deteriorations. In addition, the time-varying effects of gender over time are also disclosed. Third, applicable methodologies in survival analysis were employed in the study that permits valid tests of null hypotheses and confidence intervals to support those new study results.

This study also has a few limitations. First, the number of patients was only more than a hundred in this retrospective study, which allows examining approximately 10 factors in the models. More participants are needed if many more factors are to be tested. Second, although the designed LF events have temporality and severity meanings, they are still limited in some situations. For example, the upgrades from grade 0 to grade 1 and grade 0 to grade 2 on the same day were indistinguishably defined as an LF event that day. Third, the etiology of female participants with higher hazards for LF events is unknown. Further well-designed studies for causal inference are required to strengthen this observation.

## Conclusions

Baseline LF score before radiotherapy and gender would be significant risk factors associated with LF deteriorations within fluctuating long-term LF after hepatic radiotherapy in patients with HCC. In addition, the differences in characteristics of their effects indicate the changes in high-risk groups over time. These discoveries are beyond the scope of RILD and would help develop prevention and monitoring strategies for different groups at different time stages. Clinical outcomes and survival of HCC patients could be further improved.

### Supplementary Information

**Additional file 1: Figure S1.** Correspondence of baseline liver function (LF) score and LF event incidence for the three common liver chemistries. Specifically, individual scores (BIL, AST, and ALT scores) aligned with LF grading levels in CTCAE v5.0. The total score is the sum of individual scores, referred to as the ALL3 score, which encompasses the cumulative values of BIL, AST, and ALT scores.

**Additional file 2: Figure S2.** Correspondence of baseline liver function (LF) score and LF event incidence for the four common liver chemistries. Specifically, individual scores (BIL, AST, ALT, and ALKP scores) aligned with LF grading levels in CTCAE v5.0. The total score is the sum of individual scores, referred to as the ALL4 score, which encompasses the cumulative values of BIL, AST, ALT, and ALKP scores.

**Additional file 3: Table S1.** Numbers of liver function (LF) events according to different LF combinations.

**Additional file 4: Table S2.** Probability that the assumption of constant hazard over time was valid.

**Additional file 5: Table S3.** Hazard ratios in developing individual types of liver function events.

**Additional file 6: Table S4.** Hazard ratios in developing combined liver function events for the three and four common liver chemistries with/without introducing time-dependent covariates in the Cox models.

**Additional file 7: Table S5.** Variation in hazard ratios was minor when adjusting the cutoff values of the liver function grading scale by multiplying the cutoff values by a parameter.

## Data Availability

The data sets used and/or analyzed during the current study are available from the corresponding author on reasonable request.
