# Supplementary figures and images for: Time variation of high-risk groups for liver function deteriorations within fluctuating long-term liver function after hepatic radiotherapy in patients with hepatocellular carcinoma
Source: Eur J Med Res. 2024 Feb 7;29:104. doi: 10.1186/s40001-024-01692-z (PMC10848403; doi:10.1186/s40001-024-01692-z)

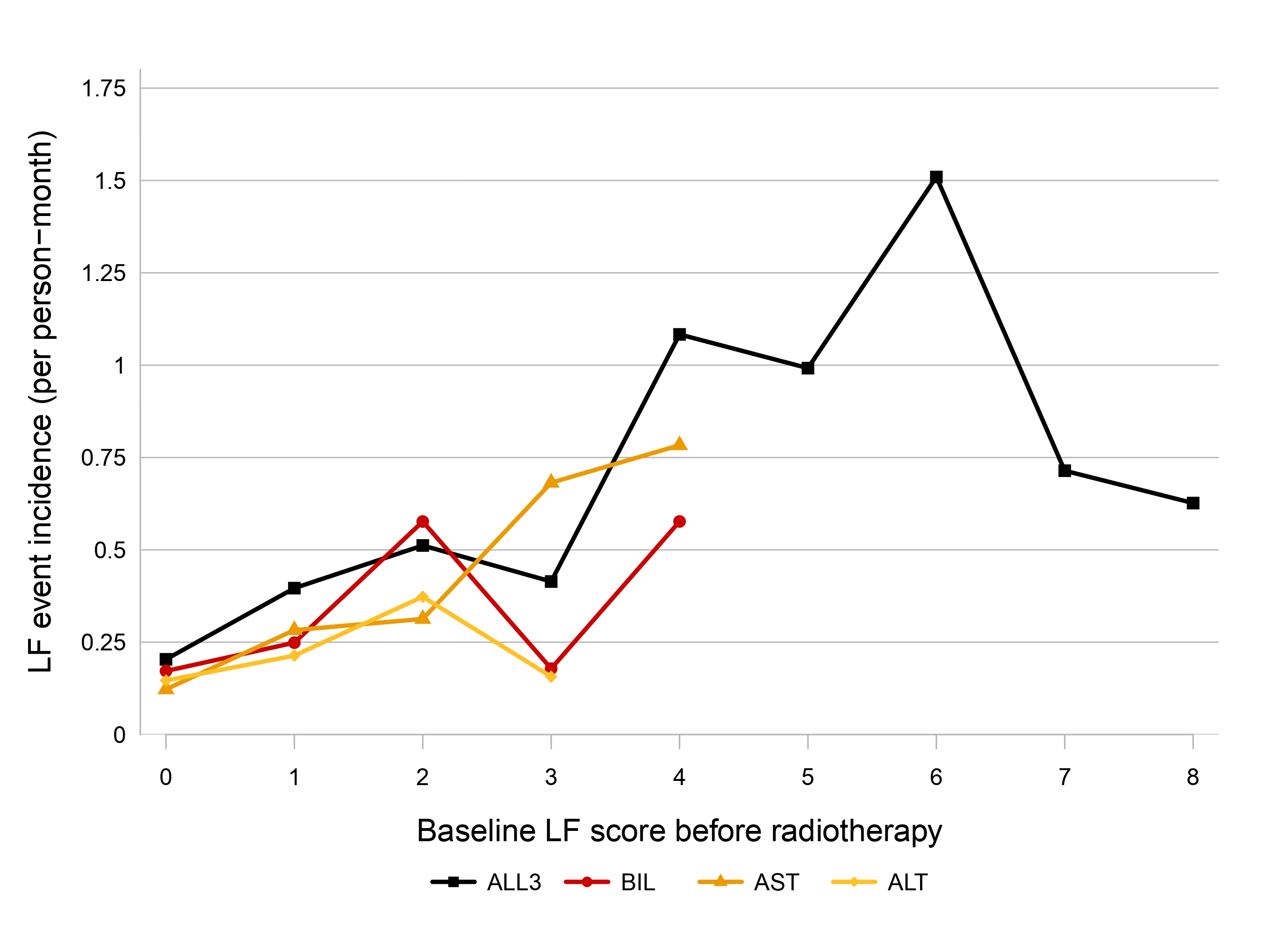

Supplement: Supplementary file 1 — Additional file 1: Figure S1. Correspondence of baseline liver function (LF) score and LF event incidence for the three common liver chemistries. Specifically, individual scores (BIL, AST, and ALT scores) aligned with LF grading levels in CTCAE v5.0. The total score is the sum of individual scores, referred to as the ALL3 score, which encompasses the cumulative values of BIL, AST, and ALT scores. [file 40001_2024_1692_MOESM1_ESM.jpg]

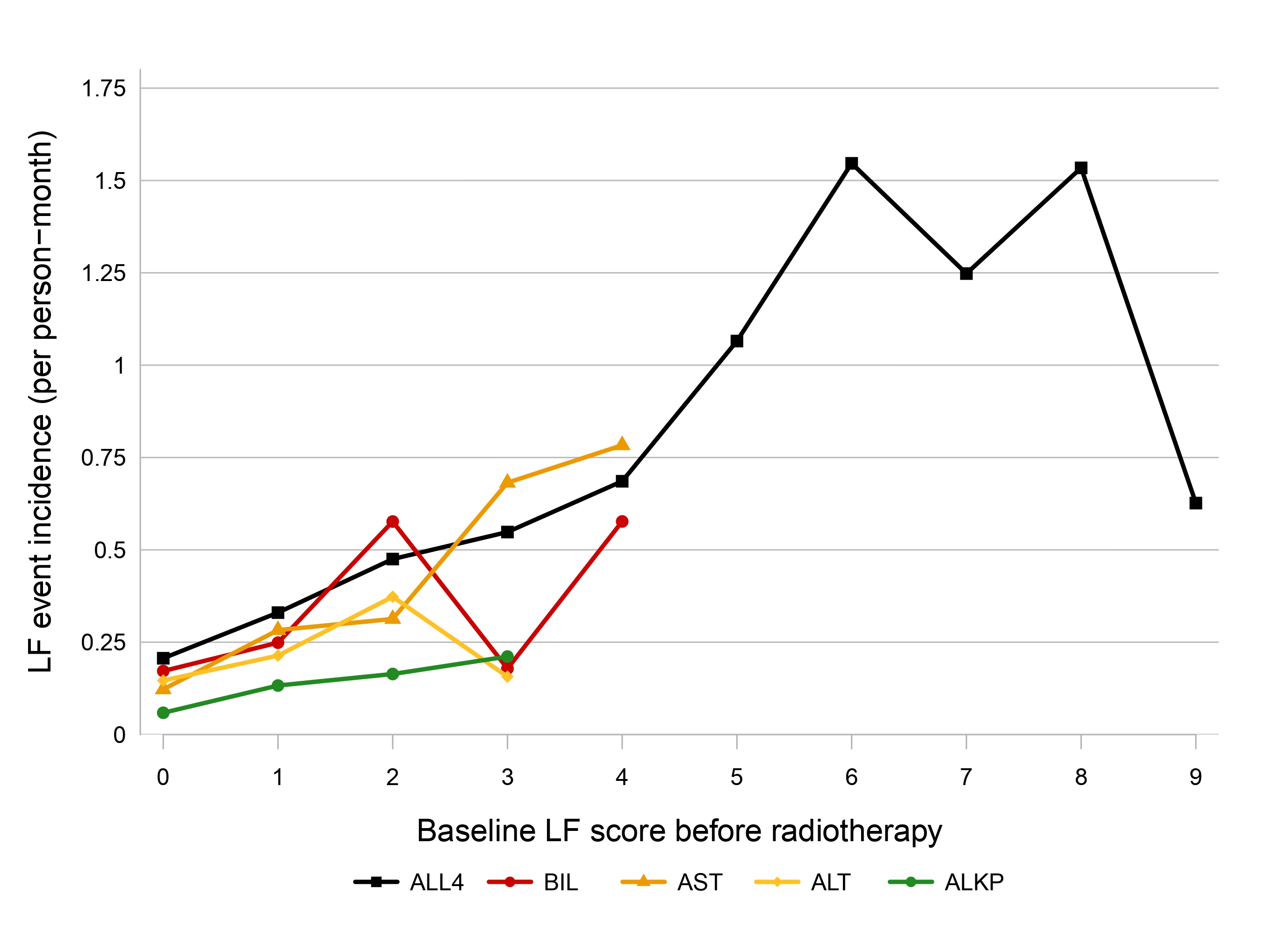

Supplement: Supplementary file 2 — Additional file 2: Figure S2. Correspondence of baseline liver function (LF) score and LF event incidence for the four common liver chemistries. Specifically, individual scores (BIL, AST, ALT, and ALKP scores) aligned with LF grading levels in CTCAE v5.0. The total score is the sum of individual scores, referred to as the ALL4 score, which encompasses the cumulative values of BIL, AST, ALT, and ALKP scores. [file 40001_2024_1692_MOESM2_ESM.jpg]
