# Supplementary material for: Time variation of high-risk groups for liver function deteriorations within fluctuating long-term liver function after hepatic radiotherapy in patients with hepatocellular carcinoma
Source: Eur J Med Res. 2024 Feb 7;29:104. doi: 10.1186/s40001-024-01692-z (PMC10848403; doi:10.1186/s40001-024-01692-z)
Supplement: Supplementary file 3 — Additional file 3: Table S1. Numbers of liver function (LF) events according to different LF combinations. [file 40001_2024_1692_MOESM3_ESM.docx]

Additional file 3: Table S1.

| No. of types | Type of liver function | No. of events |
| --- | --- | --- |
| 1 | BIL | 148 |
|  | AST | 132 |
|  | ALT | 113 |
|  | ALKP | 33 |
|  | INR | 76 |
|  | ALB | 123 |
| 2 | BIL+AST | 36 |
|  | BIL+ALT | 17 |
|  | BIL+ALKP | 7 |
|  | BIL+INR | 16 |
|  | BIL+ALB | 14 |
|  | AST+ALT | 64 |
|  | AST+ALKP | 8 |
|  | AST+INR | 10 |
|  | AST+ALB | 10 |
|  | ALT+ALKP | 4 |
|  | ALT+INR | 6 |
|  | ALT+ALB | 4 |
|  | ALKP+INR | 1 |
|  | ALKP+ALB | 5 |
|  | INR+ALB | 8 |
| 3 | BIL+AST+ALT | 40 |
|  | BIL+AST+ALKP | 4 |
|  | BIL+AST+INR | 6 |
|  | BIL+AST+ALB | 2 |
|  | BIL+ALT+ALKP | 2 |
|  | BIL+ALT+INR | 5 |
|  | BIL+ALKP+INR | 2 |
|  | BIL+ALKP+ALB | 2 |
|  | BIL+INR+ALB | 1 |
|  | AST+ALT+ALKP | 5 |
|  | AST+ALT+INR | 6 |
|  | AST+ALT+ALB | 4 |
|  | ALT+ALKP+INR | 1 |
|  | ALT+INR+ALB | 1 |
| 4 | BIL+AST+ALT+ALKP | 12 |
|  | BIL+AST+ALT+INR | 9 |
|  | BIL+AST+ALKP+INR | 1 |
|  | BIL+ALKP+INR+ALB | 1 |
|  | AST+ALT+ALKP+INR | 1 |
|  | AST+ALT+ALKP+ALB | 2 |
|  | AST+ALT+INR+ALB | 1 |
| 5 | BIL+AST+ALT+ALKP+INR | 1 |
|  | BIL+AST+ALT+ALKP+ALB | 2 |
|  | BIL+AST+ALT+INR+ALB | 1 |
| Total | – | 947 |
| *Abbreviations:* BIL = bilirubin; AST = aspartate aminotransferase; ALT = alanine aminotransferase; ALKP = alkaline phosphatase; INR = international normalized ratio; ALB = albumin. | | |
