# Supplementary material for: Time variation of high-risk groups for liver function deteriorations within fluctuating long-term liver function after hepatic radiotherapy in patients with hepatocellular carcinoma
Source: Eur J Med Res. 2024 Feb 7;29:104. doi: 10.1186/s40001-024-01692-z (PMC10848403; doi:10.1186/s40001-024-01692-z)
Supplement: Supplementary file 4 — Additional file 4: Table S2. Probability that the assumption of constant hazard over time was valid. [file 40001_2024_1692_MOESM4_ESM.docx]

Additional file 4: Table S2.

| Covariate | Chi square | Degree of freedom | *P*-value (assumption valid) |
| --- | --- | --- | --- |
| ALL6 score | 5.792 | 1 | 0.016 |
| Age | 0.945 | 1 | 0.331 |
| Gender | 8.042 | 1 | 0.005 |
| PVTT/IVCTT | 3.009 | 1 | 0.083 |
| HBV | 1.698 | 1 | 0.193 |
| HCV | 2.136 | 1 | 0.144 |
| CTV | 18.205 | 1 | <0.001 |
| NLV | 1.560 | 1 | 0.212 |
| NLD_mean_ | 8.413 | 1 | 0.004 |
| *Abbreviations:* ALL6 = bilirubin, aspartate aminotransferase, alanine aminotransferase, alkaline phosphatase, international normalized ratio, albumin; PVTT = portal vein tumor thrombosis; IVCTT = inferior vena cava tumor thrombosis; HBV = hepatitis B virus; HCV = hepatitis C virus; CTV = clinical target volume; NLV = normal liver volume; NLD_mean_ = normal liver mean dose. | | | |
