# Supplementary material for: Time variation of high-risk groups for liver function deteriorations within fluctuating long-term liver function after hepatic radiotherapy in patients with hepatocellular carcinoma
Source: Eur J Med Res. 2024 Feb 7;29:104. doi: 10.1186/s40001-024-01692-z (PMC10848403; doi:10.1186/s40001-024-01692-z)
Supplement: Supplementary file 5 — Additional file 5: Table S3. Hazard ratios in developing individual types of liver function events. [file 40001_2024_1692_MOESM5_ESM.docx]

Additional file 5: Table S3.

| Model | Covariate | Value | Hazard ratio (95% CI) | *P*-value |
| --- | --- | --- | --- | --- |
| BIL score + covariates | BIL score | 0, 1, 2, 3, 4 (cont.) | 1.69 (1.35 – 2.10) | <0.001* |
|  | Age | ≤60, >60–68, >68–76, >76 (cont.) | 0.95 (0.76 – 1.16) | 0.619 |
|  | Gender | Female (Male ref.) | 1.56 (0.99 – 2.47) | 0.055 |
|  | PVTT/IVCTT | Positive (Negative ref.) | 1.59 (0.98 – 2.60) | 0.061 |
|  | HBV | Positive (Negative ref.) | 1.45 (0.84 – 2.52) | 0.187 |
|  | HCV | Positive (Negative ref.) | 1.35 (0.71 – 2.58) | 0.359 |
|  | CTV | Per 100 mL (cont.) | 1.01 (0.97 – 1.05) | 0.728 |
|  | NLV | Per 100 mL (cont.) | 1.00 (0.97 – 1.02) | 0.778 |
|  | NLD_mean_ | Per 1 Gy (cont.) | 0.96 (0.92 – 0.99) | 0.012* |
| AST score + covariates | AST score | 0, 1, 2, 3, 4 (cont.) | 1.45 (1.16 – 1.81) | 0.001* |
|  | Age | ≤60, >60–68, >68–76, >76 (cont.) | 1.01 (0.84 – 1.20) | 0.934 |
|  | Gender | Female (Male ref.) | 1.65 (1.16 – 2.35) | 0.006* |
|  | PVTT/IVCTT | Positive (Negative ref.) | 1.50 (1.08 – 2.08) | 0.015* |
|  | HBV | Positive (Negative ref.) | 0.87 (0.59 – 1.30) | 0.506 |
|  | HCV | Positive (Negative ref.) | 0.73 (0.48 – 1.11) | 0.137 |
|  | CTV | Per 100 mL (cont.) | 0.96 (0.92 – 1.00) | 0.054 |
|  | NLV | Per 100 mL (cont.) | 1.00 (0.98 – 1.02) | 0.849 |
|  | NLD_mean_ | Per 1 Gy (cont.) | 0.98 (0.95 – 1.01) | 0.181 |
| ALT score + covariates | ALT score | 0, 1, 2, 3, 4 (cont.) | 1.40 (1.12 – 1.76) | 0.004* |
|  | Age | ≤60, >60–68, >68–76, >76 (cont.) | 0.98 (0.83 – 1.17) | 0.832 |
|  | Gender | Female (Male ref.) | 1.62 (1.16 – 2.25) | 0.004* |
|  | PVTT/IVCTT | Positive (Negative ref.) | 1.49 (1.00 – 2.22) | 0.051 |
|  | HBV | Positive (Negative ref.) | 0.72 (0.51 – 1.04) | 0.079 |
|  | HCV | Positive (Negative ref.) | 0.68 (0.47 – 1.00) | 0.051 |
|  | CTV | Per 100 mL (cont.) | 1.01 (0.97 – 1.05) | 0.584 |
|  | NLV | Per 100 mL (cont.) | 1.01 (0.99 – 1.04) | 0.390 |
|  | NLD_mean_ | Per 1 Gy (cont.) | 0.99 (0.96 – 1.02) | 0.368 |
| ALKP score + covariates | ALKP score | 0, 1, 2, 3, 4 (cont.) | 1.82 (1.35 – 2.45) | <0.001* |
|  | Age | ≤60, >60–68, >68–76, >76 (cont.) | 1.12 (0.79 – 1.59) | 0.509 |
|  | Gender | Female (Male ref.) | 1.32 (0.73 – 2.37) | 0.361 |
|  | PVTT/IVCTT | Positive (Negative ref.) | 1.07 (0.65 – 1.77) | 0.779 |
|  | HBV | Positive (Negative ref.) | 0.87 (0.37 – 2.01) | 0.739 |
|  | HCV | Positive (Negative ref.) | 1.27 (0.52 – 3.09) | 0.606 |
|  | CTV | Per 100 mL (cont.) | 1.07 (1.03 – 1.12) | 0.002* |
|  | NLV | Per 100 mL (cont.) | 1.01 (0.96 – 1.07) | 0.635 |
|  | NLD_mean_ | Per 1 Gy (cont.) | 0.97 (0.92 – 1.02) | 0.192 |
| INR score + covariates | INR score | 0, 1, 2, 3 (cont.) | 1.99 (1.28 – 3.09) | 0.002* |
|  | Age | ≤60, >60–68, >68–76, >76 (cont.) | 0.90 (0.66 – 1.22) | 0.489 |
|  | Gender | Female (Male ref.) | 2.34 (1.20 – 4.57) | 0.013* |
|  | PVTT/IVCTT | Positive (Negative ref.) | 1.40 (0.67 – 2.91) | 0.371 |
|  | HBV | Positive (Negative ref.) | 1.02 (0.48 – 2.19) | 0.959 |
|  | HCV | Positive (Negative ref.) | 0.54 (0.24 – 1.19) | 0.126 |
|  | CTV | Per 100 mL (cont.) | 1.03 (0.95 – 1.12) | 0.510 |
|  | NLV | Per 100 mL (cont.) | 0.98 (0.94 – 1.02) | 0.365 |
|  | NLD_mean_ | Per 1 Gy (cont.) | 0.95 (0.90 – 1.00) | 0.056 |
| ALB score + covariates | ALB score | 0, 1, 2, 3 (cont.) | 1.82 (1.40 – 2.36) | <0.001* |
|  | Age | ≤60, >60–68, >68–76, >76 (cont.) | 0.95 (0.77 – 1.17) | 0.615 |
|  | Gender | Female (Male ref.) | 1.13 (0.55 – 2.33) | 0.741 |
|  | PVTT/IVCTT | Positive (Negative ref.) | 2.33 (1.34 – 4.06) | 0.003* |
|  | HBV | Positive (Negative ref.) | 0.86 (0.52 – 1.41) | 0.544 |
|  | HCV | Positive (Negative ref.) | 0.67 (0.30 – 1.46) | 0.311 |
|  | CTV | Per 100 mL (cont.) | 1.01 (0.93 – 1.10) | 0.774 |
|  | NLV | Per 100 mL (cont.) | 1.00 (0.95 – 1.06) | 0.966 |
|  | NLD_mean_ | Per 1 Gy (cont.) | 0.99 (0.93 – 1.05) | 0.743 |
| *Statistical significance  *Abbreviations:* BIL = bilirubin; AST = aspartate aminotransferase; ALT = alanine aminotransferase; ALKP = alkaline phosphatase; INR = international normalized ratio; ALB = albumin; PVTT = portal vein tumor thrombosis; IVCTT = inferior vena cava tumor thrombosis; HBV = hepatitis B virus; HCV = hepatitis C virus; CTV = clinical target volume; NLV = normal liver volume; NLD_mean_ = normal liver mean dose. | | | | |
