# Supplementary material for: Time variation of high-risk groups for liver function deteriorations within fluctuating long-term liver function after hepatic radiotherapy in patients with hepatocellular carcinoma
Source: Eur J Med Res. 2024 Feb 7;29:104. doi: 10.1186/s40001-024-01692-z (PMC10848403; doi:10.1186/s40001-024-01692-z)
Supplement: Supplementary file 6 — Additional file 6: Table S4. Hazard ratios in developing combined liver function events for the three and four common liver chemistries with/without introducing time-dependent covariates in the Cox models. [file 40001_2024_1692_MOESM6_ESM.docx]

Additional file 6: Table S4.

| Model | Covariate | Value | Hazard ratio (95% CI) | *P*-value |
| --- | --- | --- | --- | --- |
| ALL3 score + covariates | ALL3 score | 0, 1, 2, 3, 4, 5, 6, 7, 8 (cont.) | 1.24 (1.13 – 1.35) | <0.001* |
|  | Age | ≤60, >60–68, >68–76, >76 (cont.) | 1.02 (0.86 – 1.21) | 0.811 |
|  | Gender | Female (Male ref.) | 1.53 (1.09 – 2.15) | 0.013* |
|  | PVTT/IVCTT | Positive (Negative ref.) | 1.34 (0.97 – 1.85) | 0.077 |
|  | HBV | Positive (Negative ref.) | 0.95 (0.64 – 1.42) | 0.818 |
|  | HCV | Positive (Negative ref.) | 0.92 (0.61 – 1.39) | 0.700 |
|  | CTV | Per 100 mL (cont.) | 0.99 (0.96 – 1.03) | 0.597 |
|  | NLV | Per 100 mL (cont.) | 1.00 (0.98 – 1.02) | 0.794 |
|  | NLD_mean_ | Per 1 Gy (cont.) | 0.97 (0.95 – 1.00) | 0.059 |
| ALL3 score + covariates + time-dependent covariates | ALL3 score | 0, 1, 2, 3, 4, 5, 6, 7, 8 (cont.) | 1.17 (1.06 – 1.29) | 0.002* |
|  | Age | ≤60, >60–68, >68–76, >76 (cont.) | 1.02 (0.86 – 1.21) | 0.805 |
|  | Gender | Female (Male ref.) | 1.17 (0.77 – 1.76) | 0.461 |
|  | PVTT/IVCTT | Positive (Negative ref.) | 1.27 (0.94 – 1.71) | 0.122 |
|  | HBV | Positive (Negative ref.) | 1.02 (0.69 – 1.52) | 0.919 |
|  | HCV | Positive (Negative ref.) | 0.95 (0.62 – 1.45) | 0.815 |
|  | CTV | Per 100 mL (cont.) | 0.99 (0.96 – 1.03) | 0.730 |
|  | NLV | Per 100 mL (cont.) | 1.00 (0.98 – 1.03) | 0.653 |
|  | NLD_mean_ | Per 1 Gy (cont.) | 0.98 (0.95 – 1.01) | 0.154 |
|  | ALL3 score × time | 0, 1, 2, 3, 4, 5, 6, 7, 8 (cont.) per 1 month | 1.01 (1.00 – 1.01) | 0.030* |
|  | Gender × time | Female (Male ref.) per 1 month | 1.03 (1.00 – 1.06) | 0.025* |
| ALL4 score + covariates | ALL4 score | 0, 1, 2, 3, 4, 5, 6, 7, 8, 9 (cont.) | 1.24 (1.15 – 1.33) | <0.001* |
|  | Age | ≤60, >60–68, >68–76, >76 (cont.) | 1.00 (0.86 – 1.17) | 0.958 |
|  | Gender | Female (Male ref.) | 1.52 (1.12 – 2.06) | 0.008* |
|  | PVTT/IVCTT | Positive (Negative ref.) | 1.25 (0.92 – 1.71) | 0.160 |
|  | HBV | Positive (Negative ref.) | 1.02 (0.71 – 1.46) | 0.919 |
|  | HCV | Positive (Negative ref.) | 1.11 (0.76 – 1.62) | 0.594 |
|  | CTV | Per 100 mL (cont.) | 0.99 (0.96 – 1.02) | 0.632 |
|  | NLV | Per 100 mL (cont.) | 1.00 (0.99 – 1.02) | 0.593 |
|  | NLD_mean_ | Per 1 Gy (cont.) | 0.97 (0.95 – 1.00) | 0.030* |
| ALL4 score + covariates + time-dependent covariates | ALL4 score | 0, 1, 2, 3, 4, 5, 6, 7, 8, 9 (cont.) | 1.17 (1.08 – 1.27) | <0.001* |
|  | Age | ≤60, >60–68, >68–76, >76 (cont.) | 1.01 (0.86 – 1.17) | 0.943 |
|  | Gender | Female (Male ref.) | 1.15 (0.78 – 1.69) | 0.471 |
|  | PVTT/IVCTT | Positive (Negative ref.) | 1.19 (0.89 – 1.59) | 0.234 |
|  | HBV | Positive (Negative ref.) | 1.09 (0.77 – 1.56) | 0.621 |
|  | HCV | Positive (Negative ref.) | 1.14 (0.78 – 1.67) | 0.497 |
|  | CTV | Per 100 mL (cont.) | 0.99 (0.96 – 1.03) | 0.661 |
|  | NLV | Per 100 mL (cont.) | 1.01 (0.99 – 1.02) | 0.540 |
|  | NLD_mean_ | Per 1 Gy (cont.) | 0.98 (0.95 – 1.00) | 0.090 |
|  | ALL4 score × time | 0, 1, 2, 3, 4, 5, 6, 7, 8, 9 (cont.) per 1 month | 1.01 (1.00 – 1.01) | 0.019* |
|  | Gender × time | Female (Male ref.) per 1 month | 1.03 (1.00 – 1.06) | 0.024* |
| *Statistical significance  *Abbreviations:* ALL3 = bilirubin, aspartate aminotransferase, alanine aminotransferase; ALL4 = bilirubin, aspartate aminotransferase, alanine aminotransferase, alkaline phosphatase; PVTT = portal vein tumor thrombosis; IVCTT = inferior vena cava tumor thrombosis; HBV = hepatitis B virus; HCV = hepatitis C virus; CTV = clinical target volume; NLV = normal liver volume; NLD_mean_ = normal liver mean dose. | | | | |
