# Supplementary material for: Time variation of high-risk groups for liver function deteriorations within fluctuating long-term liver function after hepatic radiotherapy in patients with hepatocellular carcinoma
Source: Eur J Med Res. 2024 Feb 7;29:104. doi: 10.1186/s40001-024-01692-z (PMC10848403; doi:10.1186/s40001-024-01692-z)
Supplement: Supplementary file 7 — Additional file 7: Table S5. Variation in hazard ratios was minor when adjusting the cutoff values of the liver function grading scale by multiplying the cutoff values by a parameter. [file 40001_2024_1692_MOESM7_ESM.docx]

Additional file 7: Table S5.

| Parameter | ALL6 score | ALL6 score × time | Gender | Gender × time |
| --- | --- | --- | --- | --- |
| 0.7 | 1.10 (1.04 – 1.17) | 1.01 (1.00 – 1.01) | 1.07 (0.73 – 1.58) | 1.03 (1.01 – 1.06) |
| 0.8 | 1.12 (1.06 – 1.18) | 1.01 (1.00 – 1.01) | 1.07 (0.70 – 1.64) | 1.04 (1.01 – 1.07) |
| 0.9 | 1.14 (1.08 – 1.21) | 1.01 (1.00 – 1.01) | 0.98 (0.63 – 1.51) | 1.05 (1.02 – 1.07) |
| 1.0 | 1.17 (1.11 – 1.23) | 1.00 (1.00 – 1.01) | 1.00 (0.64 – 1.56) | 1.04 (1.01 – 1.07) |
| 1.1 | 1.14 (1.08 – 1.21) | 1.01 (1.00 – 1.01) | 1.17 (0.77 – 1.78) | 1.03 (1.01 – 1.06) |
| 1.2 | 1.18 (1.11 – 1.25) | 1.01 (1.00 – 1.01) | 1.10 (0.69 – 1.77) | 1.04 (1.01 – 1.07) |
| 1.3 | 1.18 (1.10 – 1.27) | 1.01 (1.00 – 1.01) | 1.07 (0.66 – 1.74) | 1.05 (1.02 – 1.07) |
| *Abbreviations:* ALL6 = bilirubin, aspartate aminotransferase, alanine aminotransferase, alkaline phosphatase, international normalized ratio, albumin. | | | | |
